# Contemporary Challenges and Emerging Directions in the Diagnosis and Management of Vestibular Disorders: Toward an Integrated and Personalized Approach

**DOI:** 10.3390/jcm15187120

**Published:** 2026-09-14

**Authors:** Sinisa Maslovara

**Affiliations:** Department of Clinical Medicine, Faculty of Dental Medicine and Health, Josip Juraj Strossmayer University of Osijek, 31000 Osijek, Croatia; sinisamaslovara@yahoo.com

Vestibular disorders represent one of the most complex areas of contemporary clinical medicine owing to their marked heterogeneity in symptoms, diverse etiologies, and the often-limited correlation between objective findings and patients’ subjective experiences. Traditionally, vestibular diseases have been viewed primarily through the prism of balance impairment, vertigo, and vestibulo-ocular reflex dysfunction.

Contemporary neurotology is undergoing a profound paradigm shift. The traditional dichotomous approach, focused predominantly on lesion localization within the vestibular system, is gradually being replaced by an integrative model that considers balance disorders as the result of complex interactions among peripheral, central, sensory, cognitive, and emotional mechanisms. This new conceptual framework has enabled a better understanding of conditions such as vestibular migraine, persistent postural-perceptual dizziness (PPPD), and other functional vestibular disorders, representing one of the most significant advances in vestibular medicine over the past several decades [1,2].

Vestibular symptoms are not merely disorders of reflex function; they also reflect disturbances in the way individuals perceive themselves and the space around them. Functional vestibular disorders are therefore increasingly recognized as legitimate clinical entities rather than diagnoses of exclusion. This evolving perspective highlights the need for the continued development of neurotology as an interdisciplinary field integrating expertise from otorhinolaryngology, neurology, neuropediatrics, neuro-ophthalmology, psychology, psychiatry, physical and rehabilitation medicine, vestibular rehabilitation, cardiology, neuroradiology, and neurosurgery. Physiotherapists specialized in vestibular rehabilitation, as well as experts dedicated to the diagnosis and management of functional vestibular disorders, play an especially important role in this process. It is reasonable to expect that further specialization and subspecialization among professionals dealing with balance disorders will lead to the development of new organizational and educational models focused specifically on vestibular medicine.

The Special Issue “Clinical Diagnosis and Management of Vestibular Disorders” brings together studies that reflect precisely this paradigm shift. Rather than examining individual vestibular functions or disorders in isolation, the contributions included in this collection highlight the growing concept that vestibular symptoms and disorders arise from altered interactions within complex sensory, cognitive, emotional, and behavioral networks. Such an approach provides a more comprehensive understanding of clinical presentations and helps explain why similar objective findings may be associated with markedly different subjective experiences, while also reinforcing the need for integrated, multimodal, and individualized approaches to diagnosis and treatment.

Increasing evidence suggests that vestibular function is closely linked to spatial perception, emotional processing, and cognitive performance. Chronic conditions such as persistent postural-perceptual dizziness (PPPD) illustrate the complex interplay among vestibular symptoms, spatial orientation, anxiety, and body perception [3]. At the same time, growing interest in the relationship between vestibular dysfunction and cognitive decline has raised the possibility that vestibular markers may serve as early indicators of neurodegenerative diseases [4].

Another important theme emerging from the studies included in this Special Issue is the need for personalized diagnostic approaches across different age groups and clinical populations. Findings from investigations involving children with congenital sensorineural hearing loss [5], as well as comparative studies of vestibular function in pediatric and adult populations, indicate that vestibular assessment must consider developmental, age-related, and functional characteristics of individual patients. These observations further suggest that universal diagnostic strategies may not always be sufficient for the accurate evaluation of vestibular function.

Over the past three decades, vestibular medicine has undergone a remarkably rapid technological and conceptual transformation. The introduction of videonystagmography (VNG), despite initial resistance from parts of the professional community accustomed to electronystagmography, has provided several important advantages, including improved recording resolution, elimination of electrode-related artifacts, frame-by-frame video analysis in both real time and retrospective review, and reliable assessment of torsional, horizontal, and vertical nystagmus.

The introduction of the Video Head Impulse Test (vHIT) made it possible, for the first time, to assess the function of all six semicircular canals under physiologically relevant high-frequency conditions, in contrast to caloric testing, which evaluates only the horizontal semicircular canals at very low frequencies. Similarly, the development of vestibular evoked myogenic potentials (VEMPs) provided new insights into otolithic organ function, making it possible to assess nearly the entire peripheral vestibular system across different stimulus frequencies [6].

Advanced imaging techniques, including contrast-enhanced magnetic resonance imaging of the brain and cerebellopontine angles (3T MRI with FIESTA/CISS sequences), together with the development of telemedicine-based approaches, have further expanded the diagnostic capabilities available for evaluating vestibular disorders. In recent years, we have witnessed a remarkable transformation not only in vestibular diagnostics but also in vestibular rehabilitation through the development of sophisticated computerized dynamic posturography (CDP) systems and artificial intelligence (AI), virtual reality (VR), and augmented reality (AR) technologies. Thanks to these advances, it is now possible to individualize vestibular training by simulating a wide range of real-life conditions and to optimize rehabilitation outcomes through real-time feedback.

Few areas of clinical medicine have experienced such a substantial expansion of diagnostic possibilities within a single professional generation.

However, increasing diagnostic precision has simultaneously raised new questions regarding the clinical significance of isolated or selective abnormalities. It is becoming increasingly evident that individual tests rarely provide a complete answer and that the interpretation of diagnostic findings must always be placed within a broader clinical context. The integration of information obtained through different diagnostic modalities remains essential for a meaningful understanding of vestibular function and for optimal clinical decision-making, as also reflected in contemporary multimodal diagnostic algorithms for bilateral vestibulopathy [7].

This challenge is particularly evident with the increasing use of VEMP and vHIT, which has enabled the recognition of selective or apparently isolated otolithic and semicircular canal abnormalities. As highlighted by Skacik et al. [6], such findings should not necessarily be regarded as discrete disease entities, but rather as context-dependent laboratory patterns whose clinical significance depends on reproducibility, anatomical plausibility, concordance with symptoms and signs, complementary testing, and longitudinal evolution. This illustrates a broader principle of contemporary vestibular diagnostics: greater anatomical precision does not reduce the importance of clinical integration; rather, it makes such integration increasingly essential.

The studies included in this Special Issue consistently demonstrate that the future of vestibular medicine will not be defined by the search for a single superior diagnostic test, but rather by the ability to integrate data obtained from multiple modalities into clinically meaningful and individualized models of care [8]. These contributions can be grouped into several interconnected thematic areas: studies that expand our understanding of the relationship between vestibular function, cognition, spatial perception, emotional processes, and quality of life; investigations focused on developmental and age-related aspects of vestibular function, including pediatric populations and patients with congenital hearing loss; advances in diagnostic techniques, imaging, and telemedicine-based approaches; and emerging therapeutic concepts and technological innovations that are shaping the future of vestibular rehabilitation and the management of vestibular disorders.

Particularly noteworthy is the fact that several studies extend our understanding of the vestibular system beyond the traditional boundaries of neurotology, exploring its potential role in autonomic regulation, motor development, graviception, spatial anxiety, depersonalization, and broader neurological processes such as cognition. These findings further support the growing recognition that vestibular function influences a wide range of physiological and psychological domains extending far beyond balance control alone.

Several contributions focus on diagnostic innovation. One highlights the largely untapped potential of telemedicine, a field that has been evolving for decades and now represents an important step toward improving access to specialized healthcare through accurate and timely remote assessment [9]. Another demonstrates the value of high-resolution magnetic resonance imaging and three-dimensional visualization of the labyrinth in the detection of spontaneous closure of superior semicircular canal dehiscence (SSCD) [10].

The vestibular and auditory systems are closely linked not only anatomically but also functionally. Accordingly, some studies examine the functional relationship between these systems in patients with congenital hearing loss and Ménière’s disease. Collectively, these findings further emphasize the importance of multidisciplinary collaboration among otorhinolaryngologists, neurologists, audiologists, radiologists, and rehabilitation specialists.

At the same time, therapeutic approaches to vestibular disorders are undergoing substantial change. Vestibular rehabilitation has evolved from an adjunctive therapeutic intervention into one of the fundamental pillars of treatment for many peripheral and functional vestibular disorders. Originally based on the principles of adaptation, habituation, and substitution, vestibular rehabilitation has increasingly incorporated novel approaches driven by technological advances, particularly in the field of digital health. More recently, it has continued to evolve through the integration of digital technologies, virtual and augmented reality, wearable devices, and artificial intelligence into individualized rehabilitation programs [11,12,13].

Simultaneously, improved understanding of vestibular migraine, persistent postural-perceptual dizziness (PPPD), and other functional vestibular disorders has led to the emergence of multidisciplinary treatment models that combine pharmacological therapy, vestibular rehabilitation, cognitive–behavioral approaches, and patient education [14].

Successful management of functional vestibular disorders requires recognition and treatment of anxiety, hypervigilance, catastrophizing, and other maladaptive behavioral patterns, with cognitive–behavioral interventions playing an increasingly important role.

Despite the substantial progress achieved in recent years, many important questions remain unanswered. Longitudinal studies, further standardization of diagnostic protocols, and a better understanding of the natural history of selective vestibular abnormalities are still needed. At the same time, integrating emerging technologies—including digital tools, artificial intelligence, biomarkers, and telemedicine—into routine clinical practice without compromising diagnostic reliability remains a significant challenge.

In conclusion, the studies gathered in this Special Issue illustrate how vestibular medicine is gradually evolving from a discipline focused primarily on the vestibular system and spatial perception toward a broader, more integrated field encompassing cognition, emotions, development, technology, and personalized care.

Over the coming decade, progress in vestibular medicine is unlikely to arise from the isolated assessment of individual vestibular functions and tests; rather, it will depend on our ability to integrate technological advances with a deeper understanding of the patient. Artificial intelligence, wearable technologies, digital biomarkers, home-based vestibular monitoring, virtual and augmented reality, and remote vestibular assessment have the potential to fundamentally transform the way vestibular disorders are diagnosed, monitored, and treated.

Future vestibular medicine may increasingly move beyond episodic hospital-based evaluations toward continuous, personalized care supported by objective data collected in real-world environments, with the goal of improving our understanding of the individual experience of illness and enhancing patients’ quality of life.
